# Porphyria Cutanea Tarda with Constrictive Pericarditis: A Rare Association

**DOI:** 10.1155/2012/972162

**Published:** 2012-04-12

**Authors:** Kaur Jasleen, Nidhi Sharma, Jyotika Kalsy, Mughda Sharma

**Affiliations:** ^1^Department of Dermatology, Venereology & Leprosy, Sri Guru Ram Das Hospital and Sri Guru Ram Das Institute of Medical Sciences and Research, Amritsar 143001, India; ^2^Punjab Civil Medical Services, Amritsar 143001, India; ^3^Department of Dermatology, Venereology & Leprosy, Government Medical College, Amritsar 143001, India; ^4^Department of Medicine, Sri Guru Ram Das Hospital and Medical College, Amritsar 143001, India

## Abstract

Porphyria cutanea tarda(PCT) is characterised by photosensitivity and bulla formation on photoexposed parts which heals with scaring and pigmentation. PCT is frequently associated with diabetes mellitus, lupus erythematosus and hepetitis C virus infection. We are reporting and Indian patient of PCT associated with pericarditis which is very rare.

## 1. Porphyria Cutanea Tarda (PCT) with Constrictive Pericarditis A Rare Association

Porphyrias are group of inherited or acquired disease states, which are associated with deficiency in an enzyme in the metabolic pathway of heme synthesis [1]. Porphyria cutanea tarda (PCT) is the most common type of porphyria. It is characterised by photosensitivity resulting in bullae especially on sun-exposed parts. These bullae are not surrounded by erythema and rupture easily to form erosions and shallow ulcers. Lesions heal with scarring and hypo- or hyperpigmentation. Patients frequently complain of skin fragility in affected areas. There is hyperpigmentation of skin on face, neck, and hands along with hypertrichosis on face. Sclerodermatous thickening may develop on the back of neck, in the preauricular area, thorax, fingers, and scalp [2].

Diagnosis of PCT is strongly suspected on clinical grounds, but confirmation is done by Wood's lamp examination of urine specimen which shows coral-red fluorescence in PCT. Treatment of PCT involves avoidance of alcohol and phototoxic drugs which precipitate the disease. The use of barrier sunscreens such as titanium dioxide and zinc oxide may be useful.

Phlebotomy may be required in severe cases [3], otherwise oral antimalarials are effective form of treatment in the dose of 200 mg twice weekly. The remission once induced may last for several years. PCT has been frequently associated with various diseases like diabetes mellitus, lupus erythematosus, and hepatitis C virus infection.

Constrictive pericarditis (CP) is a rare condition characterized by clinical signs of right heart failure subsequent to loss of pericardial compliance. In this disease there is encasement of the heart by a rigid nonpliable pericardium due to dense fibrosis and adhesions. Constrictive pericarditis is the result of a spectrum of primary cardiac and noncardiac conditions. The top three causes of constrictive pericarditis are idiopathic (presumably viral), post-cardiothoracic surgery, and irradiation therapy, which, according to a recent study, are responsible for 46%, 37%, and 9% of cases, respectively [4]. Less common causes of CP include connective tissue disorders, porphyria, and so forth.

We report a case of 35-year old male patient who presented to medicine outpatient department of our hospital with the complain of breathlessness with exertion and oedema feet for the last two years and complain of dull aching pain in abdomen with distended abdomen since 18 months. There was no history of jaundice blood transfusion and no history of intake of photosensitising drugs. There was a remarkable history of blisters on the exposed parts of the body off and on for which he was applying some topical creams from local practitioners. Some of the lesions healed with whitish scars and there was hyperpigmentation of skin mostly on the exposed parts.

On systemic examination he was not pale no icterus, no clubbing, and no lymphadenopathy, but jugular venous pressure was raised without any collapse. His blood pressure was 110/70 mmHg and puls paradoxus was present. There was ascites, without any hepatosplenomegaly, but chest was clear and cardiovascular examination revealed ejection systolic murmur at the apex.

**Figure 1:**
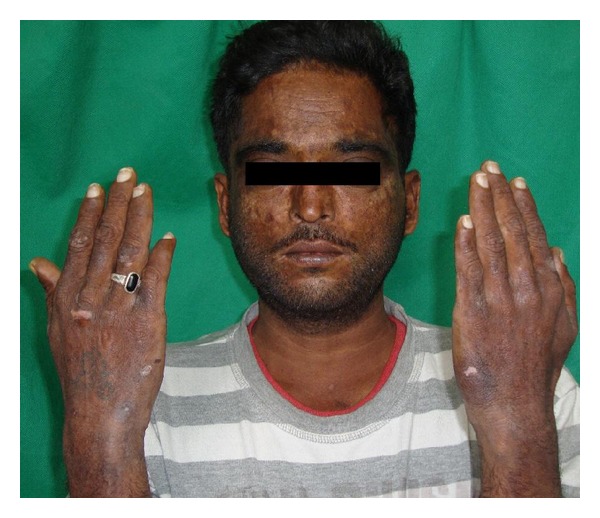
Photograph showing Hyper pigmented and atrophic whitish macules on skin on photoexposed parts.

On mucocutaneous examination, The patient was noted to have hyperpigmented skin on exposed parts of the body especially face, arms, hands, and feet. There were atrophic whitish macules seen on dorsum of hands, cheeks, and over the lips and lichenified skin at some places (Figure 1).

 Investigations complete blood count was normal but LFT showed SGOT—158, SGPT—169, and alkaline phosphate—180 with normal bilirubin. The viral markers were negative, and serum ferritin was normal. Liver biopsy was normal. ECG was normal, and on echocardiography no pericardial effusion was found, and Echo and Doppler study showed features suggestive of CP. The ultrasonography of abdomen showed gross ascites. Mantoux test was normal. ANA and double-stranded DNA were negative. His uroporphyrins in urine came out to be positive. His urine colour changed to orange on exposure to sunlight. Skin biopsy was not done.

He was given tablet Hydroxychloroquine 200 mg once a day for three months to which he responded well.

Porphyria is a very rare cause for constrictive pericarditis, and while going through literature we could find only 4 cases so far reported in the literature [5]. We report this rare association in an Indian patient.
